# Combination of Biomarkers and Novel Diagnostic Tools in the Management of Osteoporosis in Chronic Kidney Disease: An Update

**DOI:** 10.3390/jcm15124712

**Published:** 2026-06-17

**Authors:** Maria Vittoria Mollica, Giuseppe Cianciolo, Ciro Santoro, Filippo Fimognari, Olga Baraldi, Miriam Di Nunzio, Rosita Greco, Lilio Hu, Guido Di Dalmazi, Guido Zavatta, Michele Provenzano

**Affiliations:** 1 Department of Medical and Surgical Science (DIMEC), Alma Mater Studiorum—University of Bologna, 40138 Bologna, Italy; maria.mollica@studio.unibo.it (M.V.M.); olga.baraldi3@unibo.it (O.B.); miriam.dinunzio2@unibo.it (M.D.N.); guido.didalmazi@unibo.it (G.D.D.); guido.zavatta@unibo.it (G.Z.); 2 Nephrology, Dialysis and Kidney Transplant Unit, IRCCS Azienda Ospedaliero, Universitaria di Bologna, 40138 Bologna, Italy; giuseppe.cianciolo@aosp.bo.it; 3 Internal Medicine Unit, Department of Pharmacy, Health and Nutritional Sciences, University of Calabria, Rende-Hospital “SS. Annunziata”, 87100 Cosenza, Italy; ciro.santoro@unical.it; 4 Multidisciplinary Medical Department, Azienda Ospedaliera “Annunziata-Mariano Santo-S. Barbara”, 87100 Cosenza, Italy; f.fimognari@aocs.it; 5 Nephrology and Dialysis Unit, Ospedale Santa Maria delle Croci, AUSL Romagna, 48121 Ravenna, Italy; 6 Nephrology, Dialysis and Renal Transplant Unit, Department of Pharmacy, Health and Nutritional Sciences, University of Calabria, Rende-Hospital “SS. Annunziata”, 87100 Cosenza, Italy; r.greco@aocs.it; 7 Division of Endocrinology and Diabetes Prevention and Care, IRCCS Azienda Ospedaliero, Universitaria di Bologna, 40138 Bologna, Italy

**Keywords:** chronic kidney disease, osteoporosis, bone turnover markers, radiofrequency echographic multispectrometry, REMS, TBS

## Abstract

Chronic kidney disease-mineral and bone disorder (CKD–MDB) is a systemic disorder that occurs as a complication of advanced chronic kidney disease. It includes biochemical alterations (calcium, phosphorus, parathyroid hormone, vitamin D), abnormalities in bone turnover and mineralization, and vascular and soft-tissue calcifications. The development of secondary hyperparathyroidism and profound alterations in bone remodeling culminate in renal osteodystrophy, which contributes to adverse cardiovascular outcomes and increased mortality. This narrative review article aims to summarize the role of novel diagnostic techniques in the early identification of reduced bone mass and risk of fractures in patients with chronic kidney disease. The use of bone turnover markers independent of renal clearance (such as bone-specific alkaline phosphatase, procollagen type 1 N-terminal propeptide and tartrate-resistant acid phosphatase 5b) integrated with dual energy X-ray absorptiometry, trabecular bone score and radiofrequency echographic multispectrometry improves the characterization of mineral status, enabling targeted intervention to prevent bone and cardiovascular complications associated with CKD–MBD.

## 1. Introduction

At a global scale, chronic kidney disease (CKD) represents a major health burden, affecting about 14.2% of the adult population worldwide (approximately 788 million individuals) and determining a substantial prevalence of morbidity and premature mortality [1].

Alongside the progressive loss of kidney function, CKD is characterized by the early development of systemic complications that significantly influence patient prognosis, including metabolic acidosis, anemia, arterial hypertension, and significant disturbances in mineral metabolism. Among these alterations, disorders of bone and mineral homeostasis have emerged as key contributors to both cardiovascular risk and skeletal fragility. The complex constellation of abnormalities affecting mineral metabolism, bone structure, and extra-skeletal tissues in CKD is overall defined as Chronic Didney Disease–Mineral and Bone Disorder (CKD–MBD). Based on the Kidney Disease – Improving Global Outcomes (KDIGO) position statement, this syndrome encompasses a wide spectrum of biochemical alterations involving parathyroid hormone (PTH), calcium, phosphate, and vitamin D, together with impairments in bone turnover, mineralization, and volume, as well as vascular and soft-tissue calcifications. The concept of CKD–MBD has established a new paradigm of bone disease in nephrology, moving from the conventional and narrower concept of renal osteodystrophy toward a wider systemic disorder that combines skeletal pathology with cardiovascular and metabolic complications [2].

CKD-MBD manifestations may appear early during the course of kidney disease and progressively worsen with declining glomerular filtration rate, even though multiple cohort studies and meta-analyses have widely documented that even mild-to-moderate impairment in kidney function is independently linked to increased risks of cardiovascular events, hospitalization, and all-cause mortality, emphasizing the systemic nature of CKD and its complications [3,4].

From a pathophysiological perspective, disturbances in mineral metabolism reflect one of the earliest, most complex and clinically relevant complications of declining kidney function. In particular, impaired renal phosphate handling and reduced conversion of vitamin D to its active form lead to a progressive retention and alterations in calcium homeostasis, initially inducing compensatory hormonal responses that include elevated levels of fibroblast growth factor 23 (FGF23) and increased PTH secretion. Notably, FGF23 elevation has been reported to appear very early in CKD, often occurring before overt hyperphosphatemia and secondary hyperparathyroidism (SHPT), implying that adaptive endocrine changes start long before clinically apparent biochemical impairment develops [5,6]. While these compensatory responses initially intend to preserve mineral balance, their persistent activation gradually leads to SHPT and profound alterations in bone remodeling, thereby resulting in renal osteodystrophy. Within the skeletal compartment, these abnormalities result in heterogeneous patterns of bone disease, varying from low-turnover adynamic bone disease to high-turnover osteitis fibrosa, with significant consequences for fracture risk and therapeutic management. Beyond their bone effects, sustained abnormalities in phosphate and PTH homeostasis produce systemic consequences [6].

Clinical and experimental studies have shown that altered mineral metabolism and phosphate excess directly affect vascular smooth muscle cell trans-differentiation and medial vascular calcification, a sequence of events now acknowledged as a central driver of cardiovascular morbidity and mortality in CKD [7,8]. As a matter of fact, vascular calcifications in CKD are now recognized as more than a passive degenerative phenomenon, but rather as an actively regulated biological process sharing a variety of molecular pathways with physiological bone mineralization. The deposition of calcium-phosphate complexes within the vascular wall contributes to impaired vascular compliance, arterial stiffness, left ventricular hypertrophy, and increased cardiovascular mortality, especially in patients with advanced CKD and end-stage renal disease [6,7,8]. These molecular processes provide a biological explanation for the strong association between CKD–MBD and adverse cardiovascular outcomes. Among dialysis patients, higher serum phosphate levels have frequently been associated with increased mortality risk, bringing attention to the prognostic relevance of phosphate control in clinical practice [9]. Likewise, alterations in calcium and PTH concentrations have been associated with poorer outcomes, strengthening the concept that CKD–MBD biomarkers represent not only predictors of bone disease but also major determinants of overall prognosis [10,11]. According to current evidence, recent observational studies and contemporary reviews have demonstrated that abnormalities in mineral metabolism—particularly elevated PTH levels and hyperphosphatemia—are independently associated with higher risks of progression to chronic kidney disease, cardiovascular events and mortality even after adjustment for traditional risk factors [9,10]. Moreover, recent international guidelines and expert consensus statements underscore a transition from rigid biochemical targets to a personalized management of CKD–MBD, focusing on longitudinal trends, CKD stage, comorbidities, and treatment tolerability rather than isolated laboratory values [12].

Current treatment options—including dietary phosphate control, phosphate binders, vitamin D supplementation or receptor activators and calcimimetics—are increasingly implemented within a personalized approach aimed not only at managing laboratory abnormalities, but also at reducing bone and cardiovascular complications across the spectrum of CKD. In particular, the management of SHPT has developed greatly over recent years due to a better understanding of its pathogenesis and the introduction of novel pharmacological approaches able to adjust parathyroid activity and mineral metabolism more effectively [11,13].

While important advances in therapeutic options have been made over the past two decades, the optimal management of CKD–MBD still poses a significant challenge, especially in advanced stages of CKD and in patients receiving dialysis. Furthermore, the consistent variability of CKD–MBD phenotypes, as well as the limitations of current imaging techniques and available biomarkers, often adds complexity to the early identification of patients at highest risk of skeletal fragility and cardiovascular complications. Moreover, existing biochemical parameters alone may fail to adequately reflect the complexity of bone quality disorders associated with CKD, ultimately emphasizing the need for more interdisciplinary diagnostic approaches.

The aim of this review is to summarize the role of novel diagnostic techniques in the early detection of reduced bone mass in patients with chronic kidney disease. A specific focus will be given to emerging imaging modalities and circulating biomarkers in order to improve fracture risk stratification and characterization of bone turnover abnormalities. Integrating these techniques with biomarkers of bone metabolism may facilitate a more comprehensive assessment of skeletal health, enabling timely intervention and potentially reducing bone-related and cardiovascular complications associated with CKD–MBD.

## 2. Methods

A non-systematic search of the literature was conducted using PubMed, Google Scholar, and Scopus, using a combination of the following keywords: “chronic kidney disease”, “osteoporosis”, “bone turnover markers”, “radiofrequency echographic multispectrometry”, “TBS”. We restricted the research between 1 January 2000 and 1 January 2026, and we gave priority to clinical practice guidelines, systematic reviews, and original research articles published in the English language. Additional relevant publications were identified through manual screening of reference lists from articles of interest. The main aim of the study was to summarize the emerging diagnostic and therapeutic implications of imaging techniques and novel bone turnover markers in patients with CKD–MBD.

## 3. Chronic Kidney Disease: Epidemiology and Prognosis

Chronic kidney disease is considered a major global public health concern, with contemporary epidemiological analyses suggesting that its prevalence in the adult population has doubled over the last three decades (from approximately 378 million to 788 million cases). The prevalence of CKD is growing at a sustained rate together with the incidence (about 18–19 million new cases of CKD per year worldwide), primarily due to population aging and the increasing incidence of obesity, diabetes and hypertension, making CKD one of the fastest-growing non-communicable diseases and the 9th leading cause of death globally (about 1.48 million deaths worldwide in 2023).

Recent Global Burden of Disease analyses have further highlighted relevant geographic disparities in CKD prevalence and mortality, with the highest increase detected in low- and middle-income countries, where disease progression and poor outcomes are further promoted by limited access to preventive care and delayed diagnosis [1].

In addition, due to its epidemiological impact, CKD imposes a considerable socioeconomic burden on healthcare systems because of its frequent hospitalizations, the high costs related to advanced therapies such as dialysis and kidney transplantation and the chronic progressive nature of the disease, often requiring multidisciplinary care and determining a great impact on patients’ quality of life [1].

These observations are supported by large-scale population-based cohort studies showing that even mild-to-moderate impairments in kidney function correlate with a substantially increased risk of death and cardiovascular events when compared with preserved renal function. As a result, CKD has progressively emerged as a major contributor to global health loss, ranking among the leading causes of years of life lost and underscoring its strong association with premature mortality [1,3,11].

Current international clinical practice guidelines acknowledge that CKD requires an integrated evaluation of kidney function and markers of kidney damage to define an accurate prognostic assessment of the disease, reflecting the central role of these parameters in contemporary risk stratification models [14]. Accordingly, estimated glomerular filtration rate (eGFR) and albuminuria have been identified as fundamental biomarkers for CKD classification and prognostic assessment. This recommendation is based on robust evidence derived from significant collaborative analyses conducted by the Chronic Kidney Disease Prognosis Consortium, which have observed that reductions in estimated glomerular filtration rate and the presence of albuminuria independently and synergistically foresee adverse renal and cardiovascular outcomes across different populations. As a matter of fact, these associations have been documented across different ethnicities, age groups, and clinical settings, supporting the extensive reproducibility and applicability of current CKD staging systems in routine clinical practice. Furthermore, albuminuria is now considered not only as a marker of glomerular injury but also as an indicator of systemic vascular damage and generalized endothelial dysfunction, thereby explaining its strong association with cardiovascular morbidity and mortality [15,16,17].

To summarize, CKD is increasingly regarded as a systemic disorder beyond its direct renal consequences, characterized by profound metabolic, inflammatory, endocrine, and cardiovascular alterations, reinforcing the importance of early diagnosis, accurate risk stratification, and timely therapeutic intervention aimed at reducing both renal and extra-renal complications.

## 4. Bone Disorders in CKD

Bone disorders represent a key component of CKD–MBD and contribute significantly to morbidity and impaired quality of life in patients with CKD. Skeletal abnormalities are highly heterogeneous, involving alterations in bone mineralization, volume, turnover, and microarchitecture—conventionally referred to as renal osteodystrophy—as well as CKD-associated osteoporosis, a distinct but frequently overlapping clinical entity [2,8].

This broad spectrum of skeletal anomalies reflects the complex pathophysiological crosstalk between declining kidney function, disturbances in mineral metabolism, progressive alterations in bone remodeling pathways and hormonal dysregulation. According to the KDIGO classification system, renal osteodystrophy incorporates different histopathological phenotypes characterized by variable degrees of bone turnover and mineralization defects, varying from high-turnover osteitis fibrosa associated with SHPT to low-turnover adynamic bone disease [2]. More importantly, these impairments may emerge early in kidney disease and progressively worsen with declining renal function, ultimately leading to increased skeletal fragility and fracture risk, supporting the need for prompt detection and longitudinal monitoring of CKD–MBD [6].

**Figure 1:**
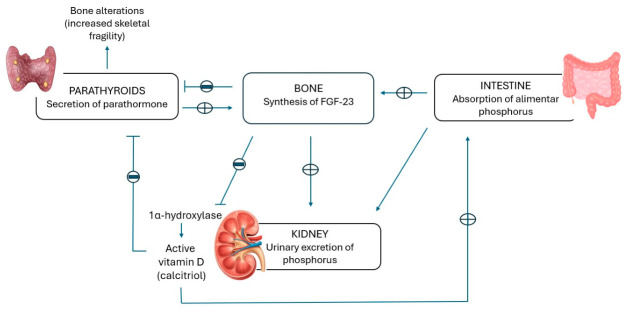
The interplay among bone, kidney, parathyroids and intestine in CKD–MBD. Synthesis of FGF-23 is stimulated by increased levels of PTH and dietary phosphorus loading. FGF-23 acts on the kidney to promote urinary phosphate excretion and suppress renal active vitamin D (calcitriol) production by inhibiting 1α-hydroxylase activity. The decreased levels of calcitriol reduce intestinal absorption of phosphorus; on the other hand, high levels of calcitriol stimulate intestinal phosphate absorption and inhibit PTH secretion. Under physiological conditions, FGF-23 also suppresses PTH secretion, whereas calcitriol exerts negative feedback on the parathyroid glands and inhibits PTH production. In advanced stages of CKD, resistance of the parathyroid glands to FGF-23 may develop, weakening the inhibitory effect of FGF-23 on PTH secretion and contributing to SHPT. Arrows with a plus sign (+) indicate an increase or upregulation of the parameter, while arrows with a minus sign (−) indicate a decrease or reduction.

The disruption of bone remodeling in CKD is driven by multiple mechanisms, encompassing impaired phosphate excretion, reduced synthesis of active vitamin D, SHPT and dysregulation of bone-derived signaling pathways involving factors such as fibroblast growth factor 23 (FGF-23) and sclerostin (Figure 1) [5,15]. Among these mechanisms, the early rise in FGF-23 concentrations and the consequent phosphate retention play a key role in the initiation of CKD–MBD, preceding the onset of hyperphosphatemia and contributing to alterations in vitamin D metabolism and PTH regulation. In particular, the early increase in circulating FGF-23 has been recognized as one of the first detectable biochemical alterations of CKD–MBD, indicating the adaptive response to phosphate retention and disturbed mineral metabolism [5]. Sustained stimulation of these compensatory pathways gradually disrupts both cortical and trabecular bone architecture, compromising bone strength and biomechanical competence. At the same time, reduced levels of active vitamin D promote not only impaired calcium absorption and SHPT but also defective mineralization and deterioration of bone quality.

In parallel, growing evidence suggests that chronic inflammation and immune dysregulation play an important role in the complex skeletal alterations observed in this population. Specifically, chronic uremic toxicity and pro-inflammatory cytokines have been reported to interfere with osteoblast and osteoclast activity, thus promoting the progression of non-remodeling alterations and leading to progressive skeletal fragility. Moreover, the underlying chronic inflammatory condition of CKD may have a negative impact on muscle mass and physical performance, indirectly increasing the risk of falls and fractures [16]. Taken together, these observations emphasize the concept of how skeletal complications in CKD transcend bone disease itself, representing a major determinant of hospitalization, disability and global health burden [18].

As underscored in recent osteoporosis research, bone fragility suggests not only reductions in bone mass but also qualitative changes in bone structure and strength, highlighting the limitations of a purely densitometric approach to fracture risk assessment. Accordingly, growing attention has been focused on the evaluation of bone quality, microarchitectural deterioration and turnover abnormalities, which may not be adequately assessed by the conventional bone mineral density measurements alone [19].

Clinically, patients with advanced CKD—particularly those undergoing dialysis—present a markedly high prevalence of low bone mineral density and osteoporosis, with reported rates higher than 40% in contemporary cohorts [17]. However, recent epidemiological studies and meta-analyses further suggest that osteopenia and osteoporosis may also be prevalent across all CKD stages, emphasizing that bone involvement is widely reported and represents a clinically relevant manifestation throughout the different stages of kidney disease [19].

Overall, fracture risk in CKD is substantially higher than in the general population and is associated with profound clinical consequences. These observations reinforce the importance of developing more comprehensive and individualized approaches for the assessment of skeletal health in CKD, integrating imaging techniques, biochemical markers and clinical risk factors in order to improve early diagnosis and therapeutic decision-making [6,18].

## 5. Osteoporosis in Chronic Kidney Disease: Prevalence and Diagnosis

In patients with CKD, osteoporosis has increasingly become a clinically relevant yet frequently overlooked comorbidity, especially in advanced CKD stages, where reduced bone strength may coexist with complex alterations of bone turnover associated with renal osteodystrophy [19,20]. From a clinical perspective, the coexistence of these conditions is highly relevant, as bone fragility in CKD cannot be viewed solely through the lens of primary osteoporosis but must be considered within the broader pattern of CKD–MBD, in which impaired bone quality, mineral metabolism abnormalities and altered remodeling activity interact with the progressive decline in kidney function.

This overlap represents a significant diagnostic challenge, as bone fragility in CKD suggests not only quantitative loss of bone mass but also qualitative changes in bone microarchitecture, which may arise prior to detectable densitometric abnormalities. Consequently, in patients with CKD, an increased fracture risk may emerge even in the absence of severely reduced bone mineral density, underscoring the need for diagnostic strategies able to capture both bone quantity and bone quality.

In a day-to-day clinical practice, dual energy X-ray absorptiometry (DXA) is still considered the most accessible and widely used tool for the evaluation of bone mineral density and has shown prognostic value for fracture risk across the spectrum of CKD, including advanced stages. In particular, recent expert consensus validates the use of DXA in CKD stage 4–5, when the results are likely to influence clinical decision-making, especially in patients with additional risk factors for osteoporosis or previous fragility fractures [21]. These findings underline the prognostic significance of fragility fractures in CKD–MBD, which should not be regarded as merely skeletal events but rather as major clinical outcomes associated with patients’ functional decline and loss of independence [20,22].

In patients with CKD stage 3–5, the prevalence of low bone mineral density (DXA T-score ≤−2.5) is reported to be about 24.5% [19], and it is correlated to a 2–3-fold increased risk of fractures; in particular, hip fractures increase mortality risk by approximately 2–3 times in this category of patients [22].

However, growing evidence from expert consensus and clinical studies reveals that DXA alone may be insufficient to comprehensively assess the multifaceted nature of bone disease in CKD, particularly in the context of altered bone turnover, since it does not provide direct information on mineralization defects, cortical porosity and trabecular connectivity, all of which may influence bone strength in CKD patients. Accordingly, increasing attention has been directed toward complementary diagnostic approaches designed to improve fracture risk stratification.

For example, the systematic assessment of vertebral fractures—frequently underdiagnosed because often clinically silent—has been shown to provide relevant prognostic information, while ancillary assessment tools such as the trabecular bone score (TBS^®^) offer indirect insights into trabecular microarchitecture beyond areal bone density measurements [23].

Advanced imaging techniques, including high-resolution peripheral quantitative computed tomography, have provided important pathophysiological insights about the prominent role of cortical porosity and trabecular disruption in CKD-associated skeletal fragility, showing that cortical impairment, rather than trabecular bone loss alone, may represent a major determinant of fracture susceptibility in CKD. However, their application remains largely confined to research settings and specialized centers [24,25].

Collectively, these considerations support a more individualized diagnostic assessment in CKD, combining densitometry with targeted imaging and clinical evaluation to more accurately identify patients at increased fracture risk, helping clinicians distinguish conditions with predominant osteoporosis from those with complex CKD–MBD-related bone alterations and guiding more appropriate therapeutic decisions. 

## 6. Osteoporosis in Chronic Kidney Disease: Novel Therapeutic Approaches

The therapeutic management of osteoporosis in CKD has undergone substantial changes from a predominantly antiresorptive approach toward a more individualized strategy, integrating fracture risk, mineral metabolism alterations and bone turnover status. In patients with CKD, skeletal fragility occurs within the broader domain of CKD–MBD, a systemic condition characterized by abnormalities in calcium, phosphate, PTH, vitamin D metabolism, bone remodeling, and extraskeletal calcifications [20,21,22,23,24,25,26,27,28,29,30]. This conceptual shift reflects the increasing awareness that fracture risk in CKD is not solely dependent on reduction in bone mineral density but rather results from the interaction between altered bone quality, abnormalities of mineral metabolism, vascular disease, chronic inflammation and the progressive decline in kidney function [21,25,27,30]. Accordingly, therapeutic decisions should not rely exclusively on bone mineral density alone but should also require a comprehensive evaluation of bone phenotypes in order to optimize treatment selection and minimize potential adverse effects [21,27,30].

CKD–MBD includes several distinct bone phenotypes, primarily classified according to bone turnover. High-turnover bone disease or osteitis fibrosa cystica is mainly driven by SHPT and is defined by markedly increased osteoclast and osteoblast activity, accelerated bone remodeling, cortical bone loss and increased fracture risk. In fact, persistent elevations of PTH promote excessive skeletal remodeling, resulting in impaired bone strength and progressive deterioration of cortical bone integrity despite, in some cases, relatively preserved bone mineral density values [21,27]. On the other hand, low-turnover or adynamic bone disease is characterized by impaired skeletal repair capacity and suppressed bone cellular activity, mostly associated with excessive PTH suppression, vitamin D analogs, overtreatment with calcium-based binders, calcimimetics, antiresorptive therapies, diabetes mellitus or aging [21,30]. The low-turnover state observed in adynamic bone disease restricts the skeleton’s ability to adapt to mechanical stress and to repair microdamage, thereby promoting skeletal fragility regardless of apparently stable biochemical parameters [21,27]. These distinct bone phenotypes have important therapeutic implications because antiresorptive agents may further inhibit bone remodeling in adynamic bone disease, eventually worsening skeletal fragility [22,25,27]. Therefore, stratifying between high- and low-turnover bone disease has become a central requirement for individualized therapeutic decision-making in advanced CKD [21,25,30].

Although bone biopsy is still considered the gold standard for the diagnosis of renal osteodystrophy, its invasive nature restricts routine clinical use [21]. As a result, growing interest has focused on non-invasive diagnostic approaches combining imaging techniques and circulating biomarkers. Bone turnover markers less influenced by renal clearance, such as bone-specific alkaline phosphatase (BSAP), procollagen type 1 N-terminal propeptide (P1NP) and tartrate-resistant acid phosphatase 5b (TRAP-5b), may provide additional information regarding skeletal remodeling dynamics and support more accurate therapeutic decisions, especially in patients for whom bone biopsy is not feasible [21,30]. As a matter of fact, persistently elevated PTH and BSAP levels generally provide evidence for high-turnover disease, whereas low PTH and low BSAP concentrations are more suggestive of adynamic bone disease. More importantly, no single biomarker is sufficiently accurate to fully characterize renal osteodystrophy, reinforcing the use of combined biochemical and imaging-based approaches [21,30]. In parallel, imaging modalities such as DXA, TBS and radiofrequency echographic multi-spectrometry (REMS) may optimize fracture risk assessment and offer complementary information regarding bone quality in CKD patients [23,31,32,33,34,35]. While DXA remains the reference technique for the assessment of bone mineral density, TBS and REMS may offer additional insights into skeletal microarchitecture and bone quality, aspects of bone strength that are not fully captured by densitometric measurements alone [23].

In recent years, the growing understanding that fracture risk in CKD reflects a complex interplay between altered bone turnover and systemic comorbidities has increasingly been reconsidered, particularly in advanced CKD, the traditional reliance on antiresorptive therapies alone [20,25,27]. This evolving awareness has prompted a reassessment of treatment paradigms originally developed for postmenopausal osteoporosis and their applicability to patients with CKD–MBD, whose skeletal abnormalities frequently differ in pathophysiology and clinical behavior [25,27].

Bisphosphonates mediate their antiresorptive effect through inhibition of osteoclast-mediated bone resorption, thereby reducing bone turnover and preserving skeletal mass. Their long half-life and selective accumulation within mineralized bone increase their anti-fracture efficacy but also raise concerns regarding excessive suppression of bone turnover in patients with adynamic bone disease and advanced CKD. As a matter of fact, their use is usually considered in selected patients with preserved or moderately impaired renal function and evidence of high bone turnover [22,25,27].

Denosumab represents an alternative antiresorptive strategy: the molecule is a fully human monoclonal antibody directed against receptor activator of nuclear factor kappa-B ligand (RANKL), a key mediator of osteoclast differentiation, activation and survival. By inhibiting the RANK/RANKL pathway, denosumab produces potent suppression of bone resorption and significant increases in bone mineral density [25,27]. Unlike bisphosphonates, denosumab is not renally excreted, making its pharmacokinetics largely independent of kidney function and theoretically expanding its applicability to patients with advanced CKD, although its use requires careful monitoring because of the increased risk of severe hypocalcemia, particularly in patients with secondary hyperparathyroidism, vitamin D deficiency or high bone turnover states. Adequate correction of vitamin D deficiency and close monitoring of calcium levels are therefore essential when denosumab is prescribed in this population [29]. The reversibility of denosumab-induced skeletal effects after treatment discontinuation represents both an advantage and a limitation, as rapid increases in bone turnover and accelerated bone loss may occur if subsequent anti-osteoporotic therapy is not implemented [25,27].

As a result, therapeutic attention has progressively shifted toward osteoanabolic and dual-acting agents, which may be better suited to overcome the qualitative deficits of bone observed in CKD. Teriparatide, a recombinant human parathyroid hormone (1–34), stimulates osteoblastic activity when administered intermittently, promoting new bone formation and improving skeletal microarchitecture. This mechanism distinguishes it from antiresorptive agents, and it may represent a particularly attractive therapeutic option in patients with low-turnover or adynamic bone disease, where stimulation of bone formation may improve skeletal remodeling, although evidence remains limited and careful patient selection is required. By enhancing osteoblastic activity and promoting new bone formation, teriparatide may partially counteract the impaired remodeling capacity characteristic of adynamic bone disease, although evidence in advanced CKD remains limited [25,27].

Romosozumab, through inhibition of sclerostin, has emerged as a particularly promising agent because of its combined anabolic and antiresorptive properties and its ability to increase BMD even in patients with mild-to-moderate impaired kidney function. This dual mechanism of action is particularly relevant in CKD, where both impaired bone formation and excessive bone resorption may contribute to skeletal fragility [26,28]. Despite these promising results, concerns regarding possible cardiovascular safety signals and the relative paucity of long-term data in patients with advanced CKD continue to limit widespread adoption and require individualized risk-benefit assessment [25,28].

This expanding therapeutic armamentarium of antiresorptive, anabolic and dual-acting therapies offers new opportunities to tailor treatment according to CKD stage, biochemical abnormalities, fracture history and the underlying skeletal phenotype [20,21,30]. Contemporary consensus statements increasingly support an individualized management strategy integrating bone turnover assessment, mineral metabolism, imaging findings, and clinical fracture risk rather than relying on uniform treatment algorithms for this heterogeneous population. Within this pattern, the goal of therapy extends beyond increasing bone mineral density alone and comprehends the prevention of fragility fractures, the optimization of bone health and the correction of CKD–MBD anomalies that contribute to adverse skeletal and systemic outcomes. Such a personalized approach is expected to play an increasingly important role as novel diagnostic tools and targeted therapeutic options become integrated into routine nephrology practice [21,25,30].

## 7. The Role of REMS in Osteoporosis: From Diagnosis to Challenging Clinical Settings

The evaluation of osteoporosis in CKD requires an integrated diagnostic approach, since bone fragility in this setting arises from a complex interplay of alterations in bone turnover, mineral metabolism and bone quality [30,36].

DXA remains the gold standard technique for bone mineral density assessment and fracture-risk stratification; however, its interpretation may be challenging in CKD, especially in the presence of alterations in cortical-trabecular bone composition, vertebral degenerative changes, vascular or extraskeletal calcifications and obesity. Furthermore, DXA provides a two-dimensional evaluation of bone mineral density and offers limited information regarding turnover status, microarchitectural deterioration and bone quality, all of which may significantly contribute to skeletal fragility in CKD patients [30,36].

In this setting, REMS has emerged as a promising ultrasound-based, radiation-free technique for the evaluation of lumbar spine and femoral neck BMD. Through the analysis of raw radiofrequency ultrasound signals rather than relying on conventional radiographic attenuation, REMS may offer complementary information on bone status and may be particularly useful for repeated assessments in outpatient settings without radiation exposure [33,34].

Current data from general osteoporosis cohorts underscore a good agreement between REMS and DXA and the ability of REMS-derived parameters to predict incident fragility fractures over longitudinal follow-up. In a 5-year prospective study of women from the general population, REMS T-scores showed fracture-discrimination performance overall comparable to DXA, supporting its potential clinical utility beyond cross-sectional body mass density assessment [31,32].

Moreover, multiple studies have reported good diagnostic performance and reproducibility of REMS measurements at both the femoral neck and lumbar spine, sustaining its potential role as a supplementary tool for fracture-risk stratification and osteoporosis evaluation [31,32,33,34].

Several recent reviews have also emphasized the advantages of REMS, including its portability, high precision and the absence of ionizing radiation, while underlining the need for broader validation across different clinical populations [33,34].

However, these study results should not be directly applied to patients with advanced CKD, in whom the evidence remains limited. In CKD-specific cohorts, especially in dialysis, early-stage data from longitudinal studies suggest that REMS may complement DXA when conventional densitometry is difficult to interpret, as they also show that REMS and DXA may not capture identical skeletal changes, particularly cortical bone loss. These observations suggest that the two techniques may provide partially distinct information regarding bone status and reinforce the concept that REMS should currently be viewed as complementary rather than interchangeable with established densitometric techniques [35].

Therefore, REMS may enrich the diagnostic pathway for CKD–MBD, but its role should also be combined with a multimodal strategy including biochemical profiling, clinical fracture-risk assessment and, when indicated, histomorphometric evaluation [30,36].

Important limitations of REMS include potential operator dependence, restricted device availability, incomplete standardization of reference thresholds comparable to those currently available for DXA, limited validation in CKD G3–G5D populations, and the lack of large prospective fracture-outcome studies specifically designed for CKD–MBD.

Future prospective studies specifically conducted in CKD and dialysis populations will be essential to determine whether REMS can improve fracture prediction, monitor treatment response and provide clinically meaningful information beyond that obtained through conventional densitometric techniques [30,35].

## 8. Beyond Bone Density: The Role of Bone Turnover Biomarkers in Osteoporosis and CKD

The growing attention to bone turnover biomarkers in CKD arises from the limited ability of conventional imaging techniques to characterize the dynamic aspects of skeletal remodeling. In this context, circulating biomarkers may help provide important information regarding the underlying turnover state and may complement imaging findings in the evaluation of CKD–MBD [30,36].

**Figure 2:**
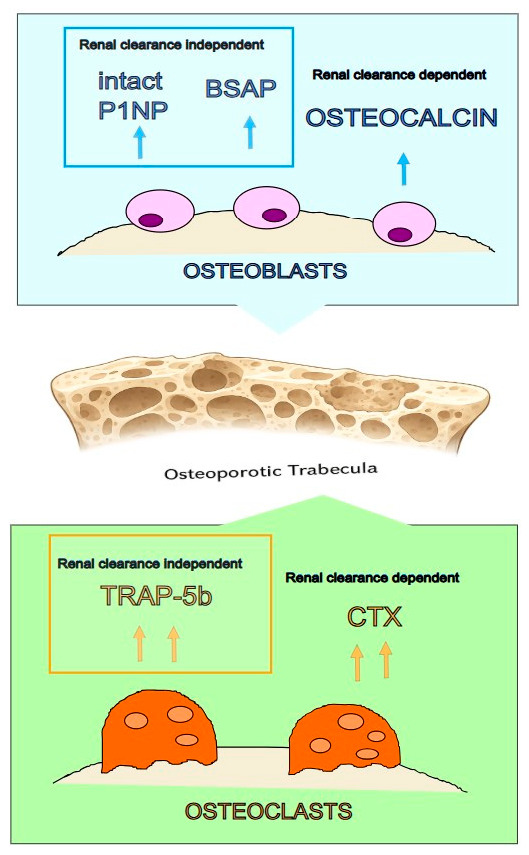
Main bone turnover markers in CKD–MBD. BSAP, P1NP, and osteocalcin reflect bone formation (osteoblasts activity), whereas TRAP-5b and CTX reflect bone resorption (osteoclasts activity). Among these, BSAP, TRAP-5b and intact P1NP are considered relatively independent of renal clearance; therefore, they are more reliable in advanced CKD.

Nevertheless, the analysis of bone turnover markers in CKD–MBD requires careful marker selection because declining glomerular filtration rate alters circulating concentrations of several molecules, potentially leading to misclassification of skeletal dynamics. Clinical interpretation must therefore consider renal clearance, laboratory techniques and parameters, biological variability and the specific clinical objective (turnover phenotyping vs treatment monitoring) [12,37,38,39,40,41,42,43,44,45,46]. In this section, the most relevant bone turnover biomarkers are discussed (Figure 2).

### 8.1. Procollagen Type I N-Terminal Propeptide

Procollagen Type I N-Terminal Propeptide (P1NP) is released during type I collagen synthesis and reflects osteoblastic activity and new bone matrix formation [37]. Because type I collagen represents the predominant organic component of the bone matrix, circulating P1NP concentrations are viewed as a direct indicator of new collagen synthesis and osteoblastic bone-forming activity, making this marker particularly useful for assessing skeletal anabolic processes [38,39].

Serum immunoassays are available in two principal formats: intact P1NP (trimeric form) and total P1NP, which also includes monomeric fragments [38,39]. One of the main challenges in the clinical interpretation of P1NP is the incomplete harmonization among commercially available assays. Differences in analytical methodology and in the molecular forms identified may result in variability across laboratories and should be acknowledged when comparing results obtained using different platforms [38,39].

Another important aspect is that P1NP should not be considered universally independent of renal clearance, since assay type substantially influences its interpretation in CKD. In advanced CKD, intact P1NP is generally preferred because total P1NP assays may detect monomeric fragments that progressively accumulate as renal function declines, eventually leading to overestimation of bone formation activity [38,39,40].

This marker may contribute, when interpreted together with BSAP and PTH, to distinguishing high-turnover states, such as secondary hyperparathyroidism, from low-turnover bone disease. However, analysis should always integrate calcium and phosphate balance, vitamin D status, total alkaline phosphatase, CKD stage and the broader clinical context, instead of relying on isolated biomarker values alone [12,40]. Moreover, although P1NP has shown associations with histomorphometric indices of bone formation, currently available evidence does not support its use as a standalone surrogate for bone biopsy when precise characterization of renal osteodystrophy is required [36,38,40].

P1NP is also extensively adopted for monitoring response to anabolic agents in osteoporosis therapy, where early increases in circulating concentrations are generally considered indicative of treatment-induced stimulation of bone formation [37,41]. Compared to static imaging techniques, P1NP provides dynamic information regarding ongoing bone formation and may therefore contribute to longitudinal assessment of skeletal remodeling over time [41,46].

### 8.2. Bone-Specific Alkaline Phosphatase

Bone-Specific Alkaline Phosphatase (BSAP) is an osteoblast-derived isoenzyme involved in bone mineralization and matrix formation by promoting hydroxyapatite crystal formation within the bone matrix [42].

Unlike several other biochemical markers influenced by reductions in glomerular filtration rate, BSAP concentrations are only minimally affected by the decline in kidney function, making it one of the most reliable bone formation markers across different CKD stages, especially in moderate-to-advanced CKD [12,40,42].

In addition, BSAP demonstrates lower biological variability than PTH in CKD populations. This characteristic may improve longitudinal assessment and facilitate the identification of clinically meaningful changes in skeletal turnover over time [42].

Nevertheless, BSAP should not be interpreted alone. Its clinical significance is strengthened when evaluated together with PTH concentrations, calcium–phosphate metabolism, vitamin D status, total alkaline phosphatase, and CKD stage, since discordant biochemical patterns may reflect mixed or evolving forms of renal osteodystrophy. Elevated BSAP levels are generally associated with increased bone turnover and secondary hyperparathyroidism, whereas low BSAP values, particularly when combined with suppressed PTH levels, may confirm the diagnosis of adynamic bone disease [12,40]. More importantly, even when PTH concentration falls within intermediate ranges, a condition in which turnover classification may be challenging, combined interpretation of BSAP and PTH has been proposed as a clinical approach to improve CKD–MBD assessment [12,40,46].

Furthermore, persistently increased BSAP concentrations have been associated with vascular calcification pathways and adverse clinical outcomes in CKD populations. These observations suggest that BSAP may reflect not only skeletal activity but also the broader systemic burden, linking abnormalities in bone metabolism with cardiovascular complications and long-term prognosis [42,43].

### 8.3. Tartrate-Resistant Acid Phosphatase 5b

Tartrate-Resistant Acid Phosphatase 5b (TRAP-5b) is an osteoclast-derived enzyme reflecting osteoclast number and activity and is considered a relatively specific marker of bone resorption. Unlike collagen degradation products, which may be substantially influenced by renal clearance, TRAP-5b is released directly by activated osteoclasts and therefore provides a more direct estimate of osteoclast burden within the skeleton [44].

Serum immunoassays selectively detect the 5b isoform [44]. TRAP-5b appears to be less affected by renal clearance and does not significantly accumulate with declining glomerular filtration rate, making it potentially useful in pre-dialysis settings, particularly when interpretation of β-CrossLaps is uncertain [41,44].

However, from a clinical perspective, as with other turnover markers, TRAP-5b should be interpreted in conjunction with PTH, BSAP, calcium, phosphate, vitamin D status, and the overall biochemical profile of CKD–MBD. Combined assessment of formation and resorption markers may improve differentiation between high- and low-turnover skeletal phenotypes [12,36,40,44].

### 8.4. C-Terminal Telopeptide of Type I Collagen

C-Terminal Telopeptide of Type I Collagen (β-CrossLaps, CTX) is a degradation fragment released during osteoclastic bone resorption. Because type I collagen constitutes the major organic component of the bone matrix, circulating CTX concentrations are largely regarded as a biochemical indicator of ongoing skeletal resorptive activity and have been extensively used in both clinical practice and osteoporosis research [37].

Serum immunoassays, including electrochemiluminescence-based methods, are commonly used for its measurement [45]. However, CTX is characterized by considerable pre-analytical and biological variability, requiring careful standardization of sample collection and processing. In particular, CTX concentrations exhibit marked circadian variation, typically peaking during the early morning hours and declining throughout the day, while food intake may further influence circulating levels. Consequently, fasting morning sampling is generally recommended to improve result reproducibility and facilitate longitudinal comparisons [37,45].

A major limitation of CTX in CKD relates to its dependence on renal clearance. CTX is predominantly eliminated through the kidneys and progressively accumulates as renal function declines, potentially leading to overestimation of bone resorption activity in moderate-to-advanced CKD. As a result, elevated CTX concentrations in patients with impaired kidney function may reflect reduced clearance rather than true increases in osteoclastic activity, thereby limiting the specificity of the marker for turnover phenotyping in CKD–MBD [40,46].

Although CTX and P1NP are internationally recommended as reference bone turnover markers for monitoring antiresorptive therapy in the general osteoporosis population [38], their interpretation in CKD requires caution and should always be contextualized according to renal function, biochemical abnormalities, and the suspected turnover pattern of CKD–MBD [40,46].

For this reason, CTX is generally considered more useful for treatment monitoring than for characterization of the underlying renal osteodystrophy phenotype. In advanced CKD, renal-independent or relatively renal-independent markers, such as BSAP, TRAP-5b, and preferably intact P1NP, are generally preferred for turnover phenotyping and non-invasive assessment of skeletal remodeling [12,40,46]. Accordingly, current CKD-MBD frameworks support the interpretation of CTX within a broader multimarker strategy rather than as a standalone indicator of bone resorption, integrating biochemical findings with mineral metabolism parameters, imaging data, and clinical context [12,46].

### 8.5. Osteocalcin

Osteocalcin is a non-collagenous protein synthesized by osteoblasts and incorporated into the bone matrix, where it contributes to the regulation of mineralization and skeletal metabolism [47]. Because it is produced during bone formation and released into the circulation, osteocalcin has traditionally been regarded as a marker of osteoblastic activity and bone formation [40,47]. Beyond its skeletal functions, osteocalcin has also attracted considerable interest as a potential endocrine mediator involved in glucose metabolism, energy homeostasis, and interactions between bone and other organ systems, highlighting the increasingly recognized role of bone as an endocrine organ [47].

Serum immunoassays detect either intact osteocalcin or various fragment forms, although substantial assay heterogeneity may limit comparability across studies and clinical laboratories [47]. The existence of multiple circulating molecular forms represents an additional challenge for clinical interpretation, as different analytical methods may quantify distinct osteocalcin fractions and therefore yield non-equivalent results [40,47].

A major limitation of osteocalcin in CKD relates to its partial dependence on renal clearance. Osteocalcin and its circulating fragments may progressively accumulate as kidney function declines, resulting in elevated serum concentrations that do not necessarily reflect increased bone formation activity [40,47]. Consequently, the relationship between circulating osteocalcin levels and actual skeletal turnover becomes increasingly difficult to interpret in moderate-to-advanced CKD, where renal retention may substantially influence measured concentrations [40]. Compared with biomarkers that are less influenced by the decline in glomerular filtration rate, such as BSAP or TRAP-5b, osteocalcin is generally regarded as a less robust tool for turnover phenotyping in CKD–MBD. Accordingly, current approaches to CKD–MBD assessment rarely rely on osteocalcin as a standalone marker and instead favor its interpretation in combination with other biochemical, imaging, and clinical information [46]. 

Below are shown the typical biochemical profiles associated with different bone turnover states in CKD–MBD (Table 1).

To summarize, the evaluation of bone turnover in CKD–MBD has progressively shifted from the interpretation of isolated biochemical alterations toward a multidimensional analysis of skeletal assets. Although multiple circulating biomarkers offer valuable information regarding bone formation and resorption, none of them is sufficiently accurate to fully characterize the complex spectrum of renal osteodystrophy when used alone. Their diagnostic performance is mainly affected by assay-related factors, biological variability and, for several markers, the progressive decline in kidney function itself [48,49,50]. This integrated approach is particularly relevant in CKD, where alterations in turnover, mineralization, bone volume, and bone quality frequently coexist and may not be adequately captured by conventional densitometric techniques, reducing the diagnostic uncertainty that often characterizes CKD–MBD [51].

Looking forward, emerging evidence in CKD–MBD studies supports the multidisciplinary approach in which biomarkers, imaging techniques and patient-specific clinical background are integrated to define skeletal phenotypes. The primary aim of strategy is to improve fracture-risk definition, enabling more targeted management in CKD–MBD through an accurate selection of antiresorptive or anabolic therapies. Other than therapeutic tailoring, recent international consensus initiatives have further emphasized the importance of bone turnover markers’ role in the treatment monitoring and longitudinal assessment of skeletal health across the continuum of CKD [48,49,50,51].

## 9. Future Perspectives and Conclusions

Skeletal complications represent a major and often underrecognized burden in patients with chronic kidney disease, reflecting the complex interaction between altered mineral metabolism, disrupted bone remodeling, and systemic comorbidities. Such complexity contributes to delayed diagnosis and suboptimal prevention of fractures when conventional diagnostic approaches are used in isolation.

In this context, innovative non-invasive techniques such as REMS may offer relevant advantages for both early diagnosis and longitudinal monitoring of osteoporosis in CKD. The absence of ionizing radiation and the feasibility of repeated assessments make REMS particularly suitable for patients requiring long-term follow-up, while preliminary data suggest its potential to detect early changes in bone properties not fully captured by standard densitometry.

The integration of advanced imaging tools with biochemical markers and clinical risk assessment may support a more personalized approach to osteoporosis management in CKD, enabling timely interventions and improved risk stratification. Ultimately, future advances are likely to arise from the combination of diagnostic innovation and individualized care strategies, with the goal of reducing fracture burden and improving long-term outcomes in this vulnerable population.
**Take Home Message**Bone disorders contribute substantially to morbidity and impaired patient quality of life in CKD.Impaired phosphate excretion, reduced synthesis of active vitamin D, secondary hyperparathyroidism, and dysregulation of FGF23 and sclerostin, together with chronic inflammation and immune dysregulation, are involved in the disruption of bone remodeling in CKD.REMS is a radiation-free ultrasound-based technique for estimating bone mineral density with the ability to capture structural features that are not fully reflected by conventional densitometry.Treatment of osteoporosis in CKD includes osteoanabolic and antiresorptive drugs; in particular, Romosozumab, with its combined anabolic and antiresorptive properties, represents a promising option.β-Cross Laps reflects osteoclastic bone resorption, while osteocalcin reflects osteoblastic bone formation; however, their use in advanced CKD is limited due to renal-dependent clearance.Bone markers independent of renal clearance include BSAP and intact P1NP for bone formation, and TRAP-5b for bone resorption. Integrating these clearance-independent markers with clinical evaluation and imaging techniques enhances bone turnover characterization, fracture risk stratification, and osteoporosis management.

## Figures and Tables

**Table 1:** Biomarker profiles and interrelationships across different bone turnover states in CKD-MBD.

*Biomarker*	* Normal Range	*Correlation with Bone Formation Markers (P1NP, BSAP, Osteocalcin)*	*Correlation with Bone Resorption Markers (TRAP-5b, CTX)*	*Low Turnover Disease*	*High Turnover Disease*
***PTH***	15–65 pg/mL (1.6–6.9 pmol/L)	Positive correlation	Positive correlation	↓ PTH associated with ↓ P1NP, ↓ BSAP, ↓ osteocalcin	↑↑ PTH associated with ↑ P1NP, ↑ osteocalcin, ↑ TRAP-5b, ↑ CTX
***FGF-23***	10–50 pg/mL	Positive correlation with osteoblastic activity	Indirect positive correlation	May be normal or mildly elevated despite low turnover	Markedly elevated; correlates with PTH and bone remodeling activity
***P1NP (intact)***	15–80 ng/mL	Strong positive correlation with BSAP and osteocalcin	Moderate positive correlation with TRAP-5b and CTX	↓↓↓	↑↑
***BSAP***	5–22 μg/L	Strong positive correlation with P1NP and osteocalcin	Moderate positive correlation with TRAP-5b and CTX	↓↓↓	↑↑↑
***Osteocalcin***	5–30 ng/mL	Positive correlation with P1NP and BSAP	Moderate positive correlation with TRAP-5b and CTX	↓↓	↑↑
***TRAP-5b***	1.2–4.5 U/L	Positive correlation with osteocalcin, P1NP and BSAP	Strong positive correlation with CTX	↓↓↓	↑↑↑
***CTX***	0.10–0.70 ng/mL	Moderate positive correlation with formation markers	Strong positive correlation with TRAP-5b	↓↓↓	↑↑↑
***Calcium***	2.15–2.55 mmol/L	Inversely related to PTH	Variable relationship	N or ↑	N or ↓ due to rapid skeletal uptake and SHPT
***Phosphate***	0.81–1.45 mmol/mol	Weak direct correlation	Indirect positive correlation through PTH/FGF23 stimulation	N or ↑	↑↑ associated with increased PTH and FGF23
***25OH vitamin D***	≥30 ng/mL	Positive association with bone formation	Indirect inverse association with excessive turnover	↓↓	↓↓ and contributed to SHPT

* Normal reference ranges in the adult population should be interpreted according to variability in laboratory assays. Upward arrows indicate an increase or elevation of the parameter, while downward arrows indicate a decrease or reduction.

## Data Availability

No new data were created or analyzed in this study.

## Abbreviations

The following abbreviations are used in this manuscript:

BMD	Bone mineral density
BSAP	Bone-specific alkaline phosphatase
CKD	Chronic kidney disease
CKD-MDB	Chronic kidney disease–mineral and bone disorder
CTX	C-terminal telopeptide of type I collagen (β-CrossLaps)
DXA	Dual energy X-ray absorptiometry
FGF-23	Fibroblast growth factor 23
GFR	Glomerular filtration rate
P1NP	Procollagen type I N-terminal propeptide
PTH	Parathyroid hormone
REMS	Radiofrequency echographic multispectrometry
SHPT	Secondary hyperparathyroidism
TBS	Trabecular bone score
TRAP-5b	Tartrate-resistant acid phosphatase 5b
